# Living Beyond IBD: A Patient’s Experience With EIMs and the Case for Multidisciplinary Care

**DOI:** 10.1093/crocol/otaf006

**Published:** 2025-01-20

**Authors:** Rocio Castrillon

**Affiliations:** Crohn’s & Colitis Foundation, New York, NY, USA

**Keywords:** Crohn’s disease, ulcerative colitis, IBD, extra-intestinal manifestations

## Abstract

This editorial explores the complex relationship between inflammatory bowel disease (IBD) and extra-intestinal manifestations (EIMs) and underscores the clinician’s critical need for comprehensive EIM care, as well as the physical and emotional burden imposed on the patient. The editorial concludes with actionable steps for clinicians and a call to advance IBD care with a comprehensive multidisciplinary approach that acknowledges the various challenges faced by patients. Together, we can transform the IBD journey into a partnership built on understanding, support, and shared hope.

As a patient living with Crohn’s disease for over 2 decades, I have come to understand and accept that my journey extends far beyond the digestive system. Like many people living with inflammatory bowel disease (IBD), my life has been shaped not only by gastrointestinal symptoms often seen with Crohn’s disease and ulcerative colitis, but also by a number of complex, extra-intestinal manifestations (EIMs) that have impacted nearly every facet of my health and my life. Living with EIMs, along with IBD, has brought me challenges beyond what I ever expected as a Crohn’s disease patient. While I have learned to adapt and my resilience has grown, the unexpectedness and severity of the EIMs I have faced are sometimes incomprehensible.

For clinicians, the invisible burden of EIMs in IBD patients may not always be apparent. These symptoms, often dismissed or overshadowed by primary gastrointestinal concerns, can quietly erode a patient’s quality of life. We may not convey and properly communicate EIMs to clinicians—either in part due to lack of knowledge, but also due to uncertainty of any correlation with IBD. Recognizing and addressing these manifestations in IBD patients is not just important—it is critical. Clinicians have the power to shift outcomes and provide relief and reassurance through early detection and coordinated, multidisciplinary care. By embracing the full spectrum of IBD, we can work together to prevent patients like me from being left in the shadows, struggling with an unseen yet profoundly impactful aspect of this disease.

The physical toll is often compounded by an emotional weight that feels unrelenting, as I try to balance EIM symptoms, alongside the daily challenges of IBD, and oftentimes suffering in silence. Every EIM that presents can bring layers of worry and unpredictability, which then affects the physical, emotional, and mental health of people living with IBD, along with affecting daily life.

Between 25% and 40% of IBD patients experience EIMs, commonly in the joints, skin, bones, eyes, kidneys, and liver (Figures 1 and 2).^1^

**Figure 1.**
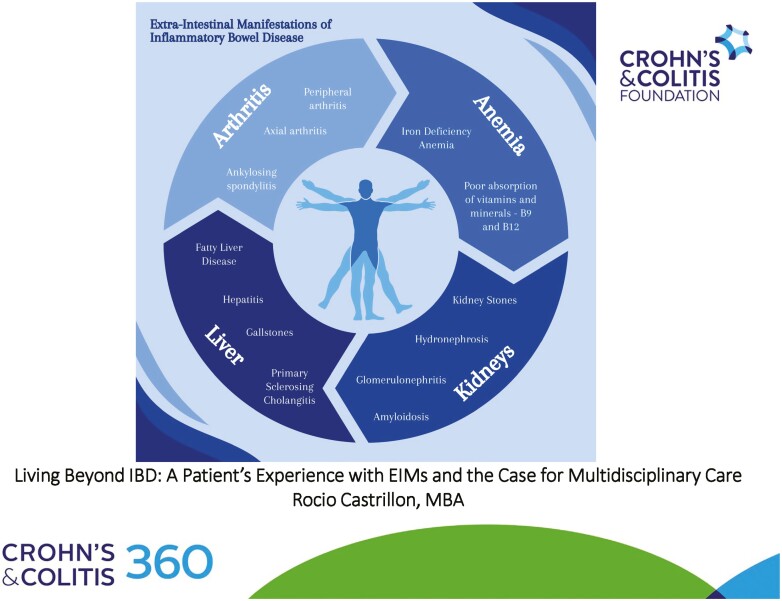
Common extra-intestinal manifestations in inflammatory bowel disease (1 of 2).

**Figure 2.**
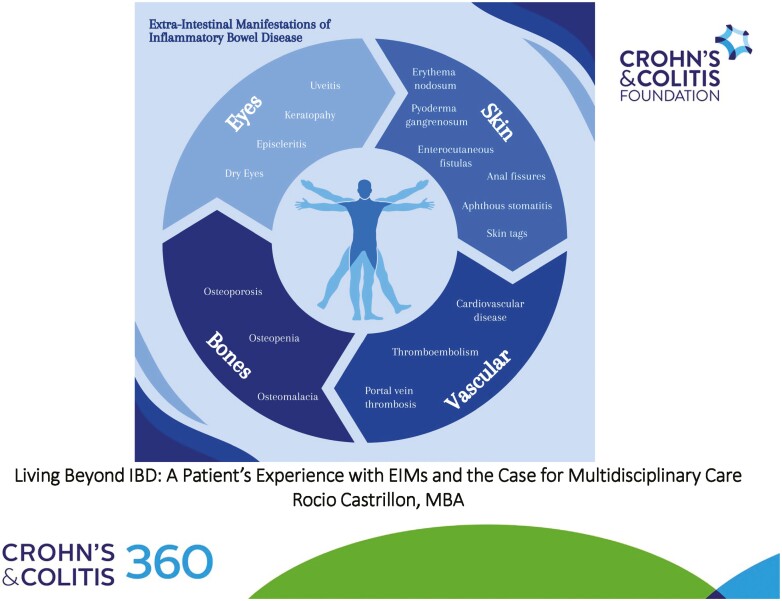
Common extra-intestinal manifestations in inflammatory bowel disease (2 of 2).

Over the years, I have been affected by a multitude of EIMs, with the most debilitating being through the initial presentation of perianal fistulae. Without a proper diagnosis for over 5 years, I underwent 2 debilitating fistulotomies, without a clear understanding as to why I did developed what could be considered both an IBD phenotype, as well as an EIM. To this day, living with fistulae have been one of the most emotionally damaging presentations of Crohn’s disease I have dealt with.

One challenging EIM I continuously battle with every single day is iron deficiency anemia which has me living in a state of exhaustion that is far beyond typical fatigue. It feels like an invisible weight pulling me down physically and mentally, making even the simplest tasks feel daunting and exhausting. It is a common and yet invisible EIM that is far from well understood and studied. When uveitis presented itself as an EIM, I never imagined I would encounter such damaging ophthalmological effects of living with IBD. The intensity of pain I have withstood while suffering through uveitis flares, along with the uveitis treatments, is unlike anything I have ever been through. Dry eyes are yet another EIM with which I live with every single day, and again while a burden on me, it is not one that is apparent to others. Despite seeking the best ophthalmological care, and utilizing the latest treatments available for dry eye, it is a frustrating EIM that does not resolve. While this demonstrates just a few EIMs I have personally encountered, it underscores the need for clinicians to not only recognize, but also rapidly address the full scope of this disease. For those of us living with these interconnected disease states, EIMs are not mere side effects; they are central to our daily struggle with IBD. Recognizing these manifestations early and approaching them with an integrated care strategy can dramatically improve patient well-being and quality of life.

For clinicians, recognizing EIMs is critical, not only because they can significantly complicate a patient’s life, but also because early detection can prevent long-term impact. With IBD, there is a strong focus solely on gastrointestinal symptoms, but oftentimes EIMs can shape a patient’s entire IBD journey. Because my Crohn’s disease presented solely through fistulae, if I had been diagnosed and treated properly, I would not have undergone 5 years of suffering in silence.

Because EIMs can be misinterpreted or dismissed, patients may suffer longer without proper treatment. Early recognition by clinicians demonstrates a keen awareness of an IBD patient’s whole body, and oftentimes early intervention can prevent the progression of the EIM. A proactive approach will benefit not only the clinician, but also the patient themselves, as they will become more aware of the presentation of EIMs. If clinicians run through a series of appropriate questions that extend beyond the gut with the patient during a visit, it allows for quick recognition of EIMs and preventative action to be taken.

Managing the complexities of EIMS, along with IBD, requires more than a single specialist can often provide. Many EIMs require specialized care that extends beyond the scope of most gastroenterology or colorectal practices. For patients who present with varying EIMs throughout the body, effective management is best achieved through a multidisciplinary approach, where healthcare providers work and collaborate together. For example, my own multidisciplinary care team extends beyond my gastroenterologist and colorectal surgeon, but also includes a primary care physician, hematologist, endocrinologist, cardiologist, dermatologist, multiple ophthalmologists, and many more (Figure 3). All of these medical providers collaborate regularly, and it allows for each to focus exclusively on the patient’s EIM symptoms. Each specialist also brings an individual perspective that contributes to a comprehensive treatment plan, which reduces patient burden.

**Figure 3.**
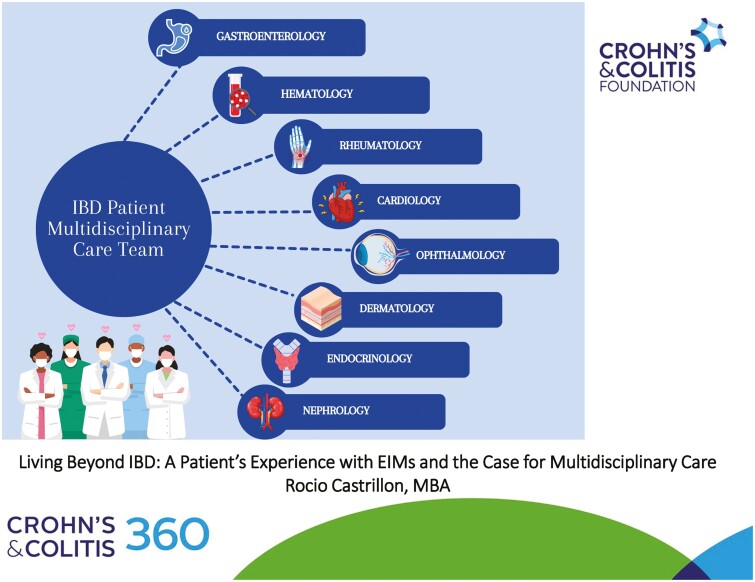
Sample inflammatory bowel disease patient multidisciplinary care team.

Because not all healthcare settings have the capabilities for a fully staffed multidisciplinary care team, it is critical to have a trusted network of colleagues with whom to consult with. When clinicians establish connections with peers across specialties, the patients benefit tremendously from a comprehensive support system of providers. It not only minimizes the burden on patients to seek out specialty care, but it allows for ongoing discussions to be had amongst clinicians to properly evaluate and treat a patient’s immediate needs.

For clinicians, one of the most crucial steps is education and staying informed about the latest research and guidelines on EIMs, as well as being keenly aware of the identification of EIMs. By incorporating routine screening for EIMs during patient evaluations, this can help identify issues early, which ultimately leads to better and quicker management. Additionally, clinicians should share resources that educate patients about the possible EIMs of IBD. If a patient is experiencing something out of the ordinary, it is beneficial to inquire with their clinician, to gain clarity and seek treatment. When patients understand the range of potential complications, they are more likely to advocate for themselves and ask relevant questions of their clinician. This collaborative approach fosters a strong relationship between patients and clinicians, and ultimately enhances the quality of care received.

In conclusion, the landscape of managing IBD extends well beyond the gut and comprehensive EIM care is integral to the patient experience. As clinicians, acknowledging the serious impact EIMs can affect patients can transform the trajectory of care. By prioritizing education, early detection, and multidisciplinary collaborative approach, IBD patients can benefit from improved quality of life. Open communication between patients and clinicians will allow for a greater understanding of a patient’s IBD and EIM symptoms and has the power to make a meaningful difference in patient outcomes.

As we move forward, I am hopeful for the strong commitment from IBD clinicians, to provide a more inclusive approach to IBD care, which recognizes and addresses the full spectrum of these diseases and EIMs. By doing so, we not only improve the lives of patients, like me, but also advance the field of gastroenterology to truly focus on a holistic model of healthcare. By ensuring no patient is left to navigate their IBD journey alone, evolving the management of IBD into a partnership can provide understanding, support, and shared hope for IBD patients.

## Data Availability

Data sharing is not applicable to this article as no new data were created or analyzed.
